# Inhibition of Gap Junctions Sensitizes Primary Glioblastoma Cells for Temozolomide

**DOI:** 10.3390/cancers11060858

**Published:** 2019-06-20

**Authors:** Anna-Laura Potthoff, Dieter Henrik Heiland, Bernd O. Evert, Filipe Rodrigues Almeida, Simon P. Behringer, Andreas Dolf, Ági Güresir, Erdem Güresir, Kevin Joseph, Torsten Pietsch, Patrick Schuss, Ulrich Herrlinger, Mike-Andrew Westhoff, Hartmut Vatter, Andreas Waha, Matthias Schneider

**Affiliations:** 1 Department of Neurosurgery, Rheinische Friedrich-Wilhelms-University Hospital, Sigmund-Freud-Strasse 25, 53127 Bonn, Germany; s4anpott@uni-bonn.de (A.-L.P.); agi.gueresir@ukbonn.de (Á.G.); erdem.gueresir@ukbonn.de (E.G.); patrick.schuss@ukbonn.de (P.S.); hartmut.vatter@ukbonn.de (H.V.); 2 Translational NeuroOncology Research Group, University of Freiburg, Breisacher Strasse 64, 79106 Freiburg im Breisgau, Germany; dieter.henrik.heiland@uniklinik-freiburg.de (D.H.H.); simon.behringer@uniklinik-freiburg.de (S.P.B.); kevin.joseph@uniklinik-freiburg.de (K.J.); 3 Faculty of Medicine, University of Freiburg, Breisacher Strasse 64, 79106 Freiburg im Breisgau, Germany; 4 Department of Neurosurgery, University of Freiburg, Breisacher Strasse 64, 79106 Freiburg im Breisgau, Germany; 5 Department of Neurology, Rheinische Friedrich-Wilhelms-University Hospital, Sigmund-Freud-Strasse 25, 53127 Bonn, Germany; b.evert@uni-bonn.de; 6 Department of Neuropathology, Rheinische Friedrich-Wilhelms-University Hospital, Sigmund-Freud-Strasse 25, 53127 Bonn, Germany; filipe.almeida@gmx.de (F.R.A.); torsten.pietsch@ukb.uni-bonn.de (T.P.); andreaswaha1@mac.com (A.W.); 7 Institute of Experimental Immunology, Rheinische Friedrich-Wilhelms-University Hospital, Sigmund-Freud-Strasse 25, 53127 Bonn, Germany; andreas.dolf@uni-bonn.de; 8 Division of Clinical Neurooncology, Department of Neurology, Rheinische Friedrich-Wilhelms-University Hospital, Sigmund-Freud-Strasse 25, 53127 Bonn, Germany; ulrich.herrlinger@ukbonn.de; 9 Department of Pediatrics and Adolescent Medicine, University Medical Center Ulm, Eythstrasse 24, 89075 Ulm, Germany; Andrew.Westhoff@uniklinik-ulm.de

**Keywords:** glioblastoma, gap junctions, INI-0602, cell death, c-Jun

## Abstract

Gap junctions have recently been shown to interconnect glioblastoma cells to a multicellular syncytial network, thereby allowing intercellular communication over long distances as well as enabling glioblastoma cells to form routes for brain microinvasion. Against this backdrop gap junction-targeted therapies might provide for an essential contribution to isolate cancer cells within the brain, thus increasing the tumor cells’ vulnerability to the standard chemotherapeutic agent temozolomide. By utilizing INI-0602—a novel gap junction inhibitor optimized for crossing the blood brain barrier—in an oncological setting, the present study was aimed at evaluating the potential of gap junction-targeted therapy on primary human glioblastoma cell populations. Pharmacological inhibition of gap junctions profoundly sensitized primary glioblastoma cells to temozolomide-mediated cell death. On the molecular level, gap junction inhibition was associated with elevated activity of the JNK signaling pathway. With the use of a novel gap junction inhibitor capable of crossing the blood–brain barrier—thus constituting an auspicious drug for clinical applicability—these results may constitute a promising new therapeutic strategy in the field of current translational glioblastoma research.

## 1. Introduction

Despite therapeutic modalities that range from surgical resection alone to surgery followed by radiotherapy with concomitant and adjuvant chemotherapy, glioblastomas continue to rank among the most disastrous and deadly tumors [1]. There are several cellular aspects that make this entity a highly lethal and difficult to treat disease. First and foremost, glioblastoma cells are characterized by a nearly unlimited proliferation capacity, marked resistance to chemotherapeutic agents, as well as fatal dissemination into healthy brain tissue [2,3,4,5].

In recent years, gap junctions as intercellular ion channels have drawn growing attention in the context of cancer pathogenesis [6]. Composed of integral membrane proteins called connexins, gap junctions allow intercellular bidirectional exchange of ions, second messengers, microRNAs, and other small molecules, therefore crucially mediating tissue homeostasis as well as cellular growth and differentiation [7,8,9]. Considering pivotal intercellular communication within a tumor’s syncytial interiority and architecture, breakdown of gap junction-based cellular co-operation has been shown to inhibit proliferation capacity as well as cellular metastatic potential in breast, melanoma and lung cancer among others [10,11,12,13]. In the context of glioblastoma biology, Osswald et al. recently revealed gap junctions—composed of two connexin-43-based hexamers—to interconnect glioblastoma cells to a multicellular network allowing intercellular communication over long distances as well as enabling glioblastoma cells to fatally invade the healthy brain tissue [14,15]. Therefore, gap junction-targeted therapies might provide an essential contribution to the isolation of glioblastoma cells from the brain. A tumor cell deprived of crucial intercellular contacts, therefore weakened to receive essential survival signals from surrounding cells, should significantly be restricted in its marked proliferation capacity, its resistance to chemotherapeutic agents, and its fatal invasive nature. 

In this context, our project was aimed at evaluating the potential of gap junction inhibition as a novel therapeutic approach in glioblastoma research. We used INI-0602, the first gap junction inhibitor that had been optimized for crossing the blood–brain barrier—an important step towards a clinical applicability, as the transition of this barrier poses an additional major challenge in glioblastoma therapy [16]. Our results show that INI-0602 profoundly sensitizes primary glioblastoma cells to temozolomide-mediated cell death. Therefore, pharmacological gap junction inhibition constitutes a promising new therapy strategy in the course of current translational glioblastoma research. 

## 2. Results

### 2.1. Characterization of Primary Human Glioblastoma Cell Populations

Three primary glioblastoma cell populations (G35, G38, and G40) were chosen from our collaborators’ collection of human brain tumor stem cells in order to gain differentiated adherent cell populations of short-term expansion for experiments [17] (see Material and Methods Section). Cells were obtained from surgical specimens of a 44-year old male, a 75-year old male, and a 57-year old female patient, as previously described [17]. The three cell populations showed significant hypermethylation in the investigated region of the MGMT gene. No mutation of the hotspot positions R132 or R172 of IDH1 and IDH2, respectively, was identified in the three cell populations (Figure S1).

### 2.2. Verification of Connexin-43 Expression in Primary Glioblastoma Cell Populations

**Figure 1:**
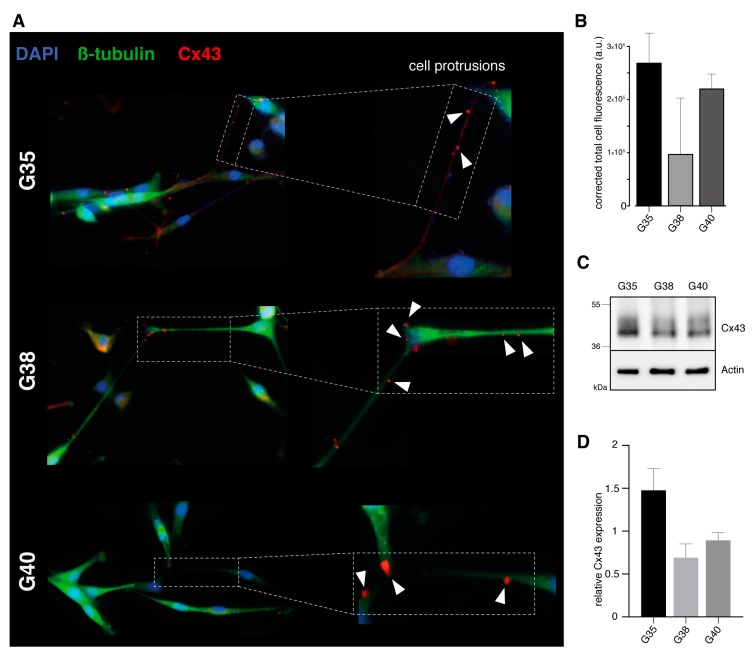
Verification of connexin-43 expression in primary glioblastoma cell populations. (**A**) Immunofluorescence staining: differentiated glioblastoma cells (G35, G38, and G40) were stained for Cx43 (red), ß-tubulin (green), and DAPI (blue). One representative image for each primary glioblastoma cell population is shown. White arrowheads point at positive Cx43 staining detected along characteristic ultra-long and lathy cell protrusions. Magnification: 40×. (**B**) Quantification of Cx43 protein expression: The relative expression level of Cx43 is shown as corrected total cell fluorescence. Mean ± SD of three measurements is depicted. (**C**,**D**) Western blot analysis and densitometric quantification of Cx43 protein expression: Actin was used as loading control. Mean ± SD of two measurements is shown. All three cell populations are positive for Cx43. G, glioblastoma; and Cx43, connexin-43.

A prerequisite for successful gap junction-targeted therapy is the evidence of target protein expression in abovementioned primary glioblastoma cell populations. Therefore, expression of intercellular gap junctions was visualized by means of immunofluorescence staining for connexin-43. All three primary glioblastoma cell populations proved to be positive for connexin-43 expression (Figure 1A,B). 

Interestingly, connexin-43 was detected along characteristic ultra-long and lathy cell protrusions that interconnected tumor cells over long distances (Figure 1A). Semiquantitative analysis revealed that the G35 primary glioblastoma population exhibits higher corrected total cell fluorescence for connexin-43 compared to G38 and G40 cell populations (Figure 1B). Western blot analysis confirmed connexin-43 protein expression for all three cell populations (Figure 1C), and reflected observed differences in the expression of relative connexin-43 (Figure 1D). Positive connexin-43 expression was also detected in primary suspension cell populations, where differentiated glioblastoma cells were derived from for short term expansion (Figure S2).

### 2.3. Determination of Effective Drug Concentrations

**Figure 2:**
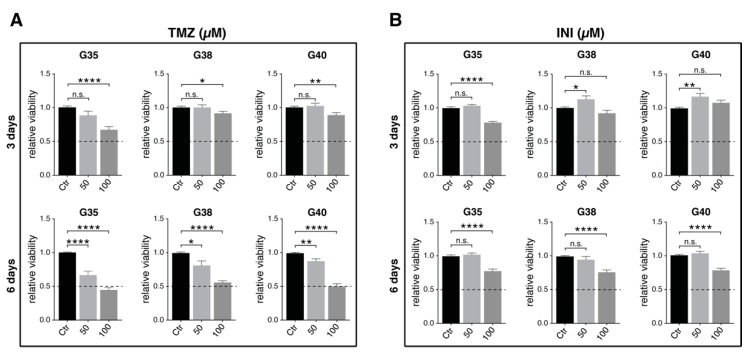
Determination of effective drug concentrations. Glioblastoma cells were treated with various concentrations of TMZ and INI. Cell viability was assessed 3 and 6 days after treatment by MTT assay (see Materials and Methods). Untreated controls were defined as 100%. Mean of relative cell viability of three independent experiments performed in triplicate are depicted. Two-tailed *t*-test was performed for comparison between different time points of treatment for concentrations of 50 µM and 100 µM of TMZ (**A**) and INI (**B**), respectively. Dotted lines represent the IC50 levels. *, ** and **** denote *p* < 0.05, *p* < 0.01, and *p* < 0.0001. G, glioblastoma; IC50, half maximal inhibitory concentration; and TMZ, temozolomide.

In order to find effective concentrations of temozolomide and INI-0602, and define significant readout time points after drug application, metabolic activity of glioblastoma cells was monitored over 144 h after treatment, as we had recently described a significant reduction in cell viability for G35, G38, and G40 cell populations 120–144 h after temozolomide treatment [18] (Figure 2A,B; for detailed information see Figure S3). In case of temozolomide, maximal inhibitory effect for all three cell populations was achieved at a concentration of 100 µM measured 144 h after treatment (Figure 2A). However, 100 µM did not correspond to a range of concentrations detected in the brain interstitium of temozolomide-treated patients [19]. Therefore, a physiologically relevant concentration of 50 µM—that could also yield a significant reduction of relative cell viability 144 h post treatment—was chosen for the following experiments.

INI-0602 exhibited only slight effects on cell viability and we could not reach conditions that corresponded to the half maximal inhibitory concentration (IC50 value) in MTT-analyses (Figure 2B). Therefore, 100 µM was taken for further analyses with regard to corresponding treatment concentrations in murine models of amyotrophic lateral sclerosis and Alzheimer’s disease [16].

### 2.4. Additional Gap Junction-targeted Therapy Significantly Diminishes Glioblastoma Cell Confluence

**Figure 3:**
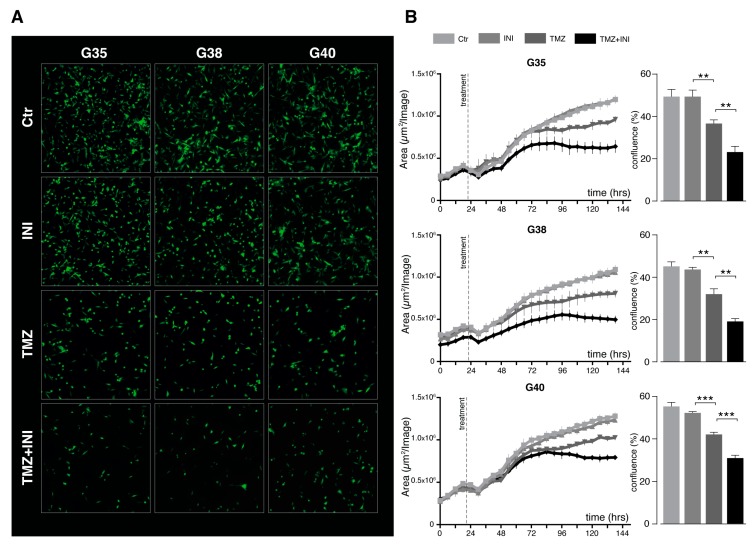
Gap junction-targeted therapy diminishes glioblastoma cell confluence. (**A**) Fluorescence images: cells were treated with 50 µM TMZ, 100 µM INI, combination of both, or left untreated. Images were taken after 6 days. One representative image out of three is shown for each cell population and treatment modality. Magnification: 10×. (**B**) Analysis of cellular confluence: quantification of fluorescence images taken with The IncuCyte^®^ S3 Live-Cell Analysis System. Cells were treated with 50 µM TMZ, 100 µM INI, combination of both, or left untreated. Cell confluence was calculated as µm^2^/Image and is depicted in 6 h intervals over a period of 6 days. Barplots to the right represent confluence in percent after 6 days for the different treatment modalities. Mean ± SD of three measurements is depicted. ** and *** denote *p* < 0.01 and *p* < 0.001. Ctr, control; G, glioblastoma; and TMZ, temozolomide.

Under the abovementioned drug concentrations and readout time points, fluorescence images revealed an expected decrease in cellular confluence for temozolomide treatment compared to untreated control (Figure 3B). While INI-0602 did not seem to markedly affect glioblastoma cell confluence, cell density was profoundly weakened for combination treatment compared to the standard chemotherapeutic agent temozolomide measured at day 6 (Figure 3A). Live cell imaging over a time span of 144 h confirmed these observations, and analysis of cellular confluence at day 6 yielded significantly diminished values for additional gap junction inhibition compared to temozolomide single treatment for all three cell populations (24.3% versus 36.8% averaged over all three primary cell populations; untreated control 49.7%; *p* < 0.01) (Figure 3B).

### 2.5. INI-0602 Sensitizes Glioblastoma Cells to Temozolomide-mediated Cell Death

**Figure 4:**
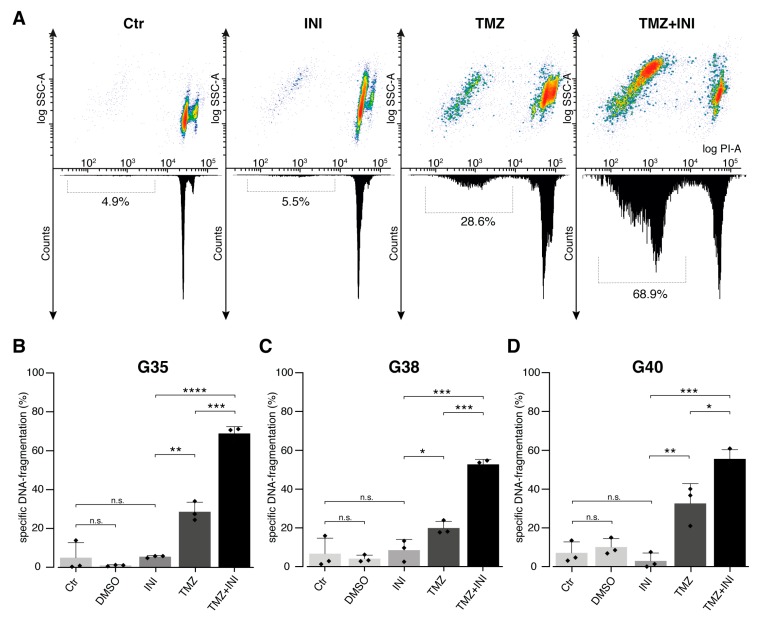
Gap junction-targeted therapy sensitizes glioblastoma cells to temozolomide-mediated cell death. (**A**) Effects on cell death: Treatment was performed with 50 μM TMZ, 100 µM INI, or combination of both. Percentage of DNA-fragmentation of propidium iodide-stained nuclei was determined by flow cytometric analysis 144 h after treatment. Representative density plots and histograms are shown for different treatment modalities for G35 cell population. The SubG1 peak is accentuated within the histograms and the mean percentage of DNA-fragmentation is depicted below. (**B**) Mean ± SD of specific DNA-fragmentation is shown for G35 (**B**), G38 (**C**), and G40 (**D**) primary glioblastoma cell populations. For each cell population three independent experiments were performed in triplicates. INI sensitizes glioblastoma cells to temozolomide-mediated cell death. Among the three glioblastoma cell populations, G35 exhibited the highest DNA-fragmentation rates. *, **, ***, and **** denote *p* < 0.05, *p* < 0.01, *p* < 0.001, and *p* < 0.0001. Ctr, control; G, glioblastoma; TMZ, temozolomide; PI, propidium iodide; and SSC, Side Scatter.

In order to characterize the underlying mechanisms of the observed effects of gap junction inhibition on cellular confluence, specific DNA-fragmentation of propidium iodide-stained nuclei was assessed as readout for cell death. Mere gap junction inhibition did not significantly increase the percentage of specific DNA-fragmentation compared to untreated control populations, however, additional administration of INI-0602 profoundly increased DNA-fragmentation rates seen for temozolomide single treatment from 27.1% up to 59.1% (*p* < 0.0001) (Figure 4A,B). Notwithstanding sensitization to temozolomide-mediated cell death was markedly present for all three cell populations, G35 and G38 primary glioblastoma cells exhibited more than a doubling of DNA-fragmentation rates compared to temozolomide alone (Figure 4C). Sub G1 peak as surrogate for cell death was highest for G35 cell population (Figure 4).

Based on the DNA-fragmentation rates the Bliss independence equation was used to investigate the interaction between TMZ and INI-0602. Bliss synergy was found for the combination treatment of TMZ and INI-0602. The observed drug effects in case of combination treatment were up to 7.3-times higher than the expected effects under the Bliss independent zero-interaction hypothesis (G35: 6.6-times higher, G38: 7.3-times higher, G40: 4.6-times higher).

Taken together, INI-0602 showed a significantly sensitizing effect for temozolomide-mediated cell death.

**Figure 5:**
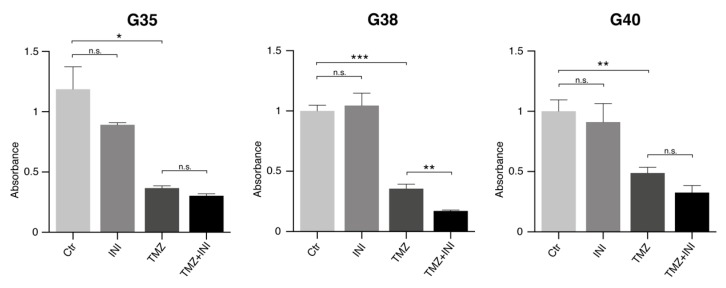
Gap junction-targeted therapy did not markedly affect glioblastoma cell proliferation. Treatment was performed with 50 μM TMZ, 100 µM INI, or combination of both. The amount of incorporated BrdU as surrogate readout for cell proliferation was determined by measuring the absorbance at 370 and 492 nm 144 h after treatment. Mean ± SD is depicted. *, ** and *** denote *p* < 0.05, *p* < 0.01 and *p* < 0.001. Ctr, control; G, glioblastoma; and TMZ, temozolomide.

As evaluation of cellular confluence alone (Figure 3) does not adequately represent cell proliferation with regard to changing cell shape and size under treatment, cell proliferation analysis was performed. Similarly to the findings deduced from analysis of DNA-fragmentation rates, single gap junction inhibition did not significantly affect glioblastoma cell proliferation compared to untreated control populations (Figure 5, for effects on cell viability see Figure S4). While temozolomide yielded expected proliferation-inhibiting effects in all three primary cell populations, combination treatment demonstrated further significant reduction of cellular proliferation only in case of G38 cell population (*p* < 0.01).

### 2.6. Additional Gap Junction Inhibition Leads to Elevated Activation of the JNK Signaling Pathway

**Figure 6:**
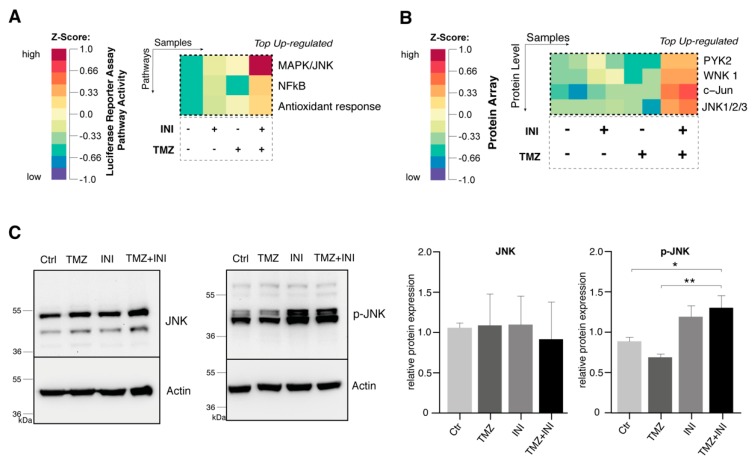
Additional gap junction inhibition leads to elevated activation of the JNK signaling pathway. (**A**) Gene expression heatmap of Cignal^TM^ Finder 45-pathway Reporter Gene Array: treatment was performed using 50 μM TMZ, 100 µM INI, or combination of both. Activity of firefly and Renilla luciferases were measured 72 h after treatment. Data were normalized for activity of Renilla luciferase to account for transfection efficiency. Experiments were performed in duplicates. Top upregulated pathways for combined treatment of TMZ and INI are displayed for G35 cell population. (**B**) Heatmap of Proteome Profiler Human Phospho-Kinase Array: cells were treated with 50 μM TMZ, 100 µM INI, or combination. Experiments were performed in duplicates. Top upregulated transcription factors and protein kinases are depicted for G35 cell population. (**C**) Western blot analysis and densitometric quantification of JNK and p-JNK protein expression. Actin was used as a loading control. Mean ± SD of relative protein expression is depicted. Experiments were performed in triplicate. Ctr, control; and TMZ, temozolomide. * and ** denote *p* < 0.05 and *p* < 0.01.

Utilizing the Cignal Finder Reporter Array, 45 oncologically relevant cell signaling pathways were assessed through reverse transfection of preformed reporter constructs into G35 primary human cell population. Measurement of luciferase activities revealed NF-kappaB as well as Activator Protein-1 (AP-1)—the major transcription factor for the JNK pathway—to be the most upregulated for combination treatment compared to single administration of INI-0602 or temozolomide (Figure 6A, for detailed information see Figure S5A).

On the protein level, c-Jun and JNK1/2/3 could be confirmed as the top up-regulated transcription factors and protein kinases in case of combination versus single drug treatment (Figure 6B, for detailed information see Figure S5B). Western blot analysis demonstrated elevated levels of p-JNK for single gap junction inhibition therapy as well as combination treatment, whereas unphosphorylated JNK did not markedly differ between untreated control populations and the treatment strategies depicted (Figure 6C).

## 3. Discussion

Gliobastoma cells have been recently reported to exhibit ultra-long and thin membrane protrusions that extend into the surrounding brain and tumor tissue in order to interconnect tumor cells over long distances. On a microscopical level, these intercellular contact points are composed of connexin-43-based gap junctions enabling glioblastoma cells to fatally assemble to a multicellular functional network [14]. The resulting syncytial structures have been shown to significantly contribute to mechanisms of therapy resistance as well as to facilitate glioblastoma invasion and proliferation capacity [15]. Therefore, we hypothesized that the inhibition of intercellular communication via gap junctions might constitute a promising novel therapeutic approach. By utilizing the first blood–brain barrier-permeable gap junction inhibitor in an oncological setting, we aimed to evaluate the potential of gap junction inhibition in the context of current translational glioblastoma research.

With regard to various studies that have observed an inverse association of gap junction expression level and glioma grade as well as less connexin-43 expression in high grade gliomas compared to normal brain tissue [20,21], we validated connexin-43 expression in three primary glioblastoma cell populations. Consistent with the recent observations of a gap junction-based multicellular syncytial network [14,15], connexin-43 expression was confirmed for all of the three glioblastoma cell populations used. In addition, positive connexin-43 staining was visualized along thin and lathy cell protrusions that interconnected glioblastoma cells over long distances, therefore providing a clinically relevant setting of human glioblastoma cell populations in order to address pivotal issues of gap junction inhibition as a novel therapeutic strategy for glioblastoma.

Current available studies on pharmacological gap junction inhibition in glioblastoma are mainly based on the use of the glycyrrhetinic acid-derivative carbenoxolone as a potent gap junction blocker [22,23,24,25,26,27]. However, carbenoxolone has been shown not to reach therapeutically relevant concentrations for gap junction inhibition in the brain interstitium after systemic administration [28]. Considering the high concentrations of carbenoxolone of about 50–100 µM that are needed in order to significantly impede intercellular communication via gap junctions in glioblastoma, measured concentrations of carbenoxolone of maximal 1µM in the brain tissue of rats are far from therapeutically required. With regard to an impeded function of the blood–brain barrier induced by the tumor itself, carbenoxolone partly could yield sensitizing effects to TRAIL-induced apoptosis in murine models of glioblastoma [22]. However, today’s requirements for sufficient additional strategies in glioblastoma therapy include the selection of drugs that harbor the capability to effectively penetrate the brain interstitium in order to reach areas with already consisting tumor infiltration, but absent blood–brain barrier impairment and contrast medium-absorbance in magnetic resonance-guided imaging. Therefore, there is a need for alternative pharmacological gap junction inhibitors that should allow for this capability. In the present study, INI-0602 as the first gap junction inhibitor optimized for crossing the blood–brain barrier that had been successfully used in murine models of amyotrophic lateral sclerosis (ALS) and Alzheimer’s disease (AD) among others [16,29,30], was evaluated in a clinically relevant human oncological setting. Thereby, gap junction inhibition has been shown to significantly sensitize glioblastoma cells to temozolomide-mediated cell death. Compared with this, proliferation-inhibiting effects could only be detected in case of one primary glioblastoma population. On the molecular level, gap junction inhibition was associated with elevated activation of the JNK as well as the NF-kappaB signaling pathway.

Compared to a considerable number of reports that initially suggested connexins to primarily function as a tumor suppressor in several cancers like tumors of the thyroid, liver, andstomach, our results are consistent with recent evidence of connexins to rather promote tumorigenesis and cancer metastasis [31,32,33]. Along these lines, gap junctions have been implicated in the intra- and extra-vasation of melanoma cells through gap junction formation between tumor and endothelial cells [10]. Furthermore, connexin-43-mediated gap junction coupling has been linked to the capability of breast cancer cells to metastasize into the healthy brain tissue [34]. Recently, breast and lung cancer cells have been shown to use connexin-43-based gap junctions in order to induce a paracrine loop with surrounding cells via transfer of the second messenger cGAMP. As a result the STAT1 and NF-kappaB pathways were activated in tumor cells thereby facilitating tumor growth and chemotherapy resistance [13]. Compared with this, effects of gap junction inhibition on cancer cell proliferation still remain a pivotal issue to be addressed. Considering this, connexins have been implicated both in promoting and decreasing tumor cell proliferation capacity [14,35,36]. These apparently contradictive findings might partly be driven by differing connexin expression levels within a tumor’s multicellular syncytium as well as reflect tumor microenvironmental disparities depending on the tumor entity studied [6,37,38]. In terms of glioblastoma biology pharmacological gap junction inhibition by carbenoxolone yielded additive effects on survival rates in temozolomide-treated orthotopic xenograft models in mice and a study of Munoz et al. could link resistance to standard chemotherapeutic agent temozolomide to intercellular connexin-43-mediated gap junction communication [27,39]. These findings support the idea of a connexin-43-based multicellular syncytium whereby glioblastoma cells might be protected from therapy-induced cytotoxic effects. Though the underlying molecular mechanisms are far from understood, recent studies could reveal pro-survival small noncoding RNA to be transferred via intercellular gap junctions resulting in increased resistance of brain metastatic lung cancer cells to chemotherapeutic agents [40,41]. Furthermore, gap junction inhibition might lead to a breakdown of intracellular homeostasis of gap junction-permeable small molecules such as Ca^2+^ thereby increasing the tumor cell’s susceptibility to chemotherapeutic agents [15]. Current available data suggest this susceptibility to be reflected by an activation of the NF-kappaB pathway as well as the JNK signaling system on a molecular level [13,42]. In line with our results of the JNK pathway to constitute the most upregulated signaling cascade for the proposed novel gap-junction based treatment strategy, the oncogene c-Jun has been described as a pivotal player within the so-called ‘Cx43 interactome’ [37]. According to this, the JNK signaling system exhibits among the strongest confidence score prediction value among connexin-43 related pathway activation and might therefore reflect main molecular insights in microscopical visible treatment strategies of the inhibition of intercellular communication via gap junctions. In the current literature, JNK signaling is controversially discussed as both pro- and anti-tumorigenic [43,44]. Along these lines, loss of JNK1 or JNK2 has been linked to elevated mammary tumor development, while impaired tumor cell proliferation and cancer development in hepatocellular carcinoma models, for example [45,46,47,48]. It appears that the balance between pro- and anti-apoptotic cellular signaling inputs finally determines the cells’ commitment to proliferation or programmed cell death [44,49]. With regard to our findings of pAKT and pERK to be downregulated under gap junction inhibition (see Figure S5B), JNK might be activated as a cellular survival strategy by NfkappaB in order to compensate impeded AKT and ERK signaling. However it has to be mentioned critically, that affected pathway activation was identified by the use of a Reporter gene assay as well as a Proteome array. As mere screening methods validity may be limited by the initial selection of individual transcription factors examined that certainly cannot reflect the variety of transcription factors and protein kinases within a particular oncological signaling pathway, and therefore might lead to initial exclusion of affected signaling pathways with regard to subsequent examination. Further investigation will be needed to identify the exact underlying mechanisms of JNK signaling involvement in the case of gap junction inhibition in glioblastoma biology—among them, whether JNK might be activated as a cellular mechanism of response to or rather reflect a direct molecular consequence of the inhibition of gap junctions.

## 4. Materials and Methods

### 4.1. Cell Culture and Lentiviral Transduction

After patients’ informed consent was obtained, primary human glioblastoma stem cell-like cells were isolated by mechanical disaggregation from surgical specimens obtained from three patients with WHO IV glioma (G35, G38 and G40) as previously described [50]. The resulting primary suspension cell populations were cultured in DMEM/F-12 (HAM) medium (Gibco, ThermoFisher Scientific, Waltham, MA, USA), supplemented with EGF (Biomol GmbH, Hamburg, Germany), FGF (Miltenyi Biotec GmbH, Bergisch Gladbach, Germany) and B27 (Gibco), and served as a stable cell pool [51]. Subsequently, suspension cells were provoked into differentiation by letting them adhere in a serum-enriched environment consisting of uncoated cell culture material in the presence of DMEM (Gibco), supplemented with 10% FCS (Gibco) and 1% Penicillin/Streptomycin (Gibco). Differentiated cell populations were maintained for no more than twelve cell passages [51].

For the fluorescence and live imaging experiments, glioblastoma cells were transduced with lentiviral particles (rLV.EF1.Zsgreen1-9, Clontech, Mountain View, CA, USA). Briefly, suspension cells were seeded into petri dishes (Falcon, Corning Inc., Corning, NY, USA) with a density of 5e4/mL. After incubation overnight (37 °C, 5% CO_2_), the medium was changed and a mixture of 5 µL Polybrene^®^ (Santa Cruz Biotechnology, Dallas, TX, USA) and 5 µL virus was added to the cells. Efficacy of transduction was measured after two days. Then medium containing lentiviral particles was discarded and cells were transferred into cell culture flasks (Falcon, Corning, Inc., Corning, NY, USA) containing normal growth medium.

### 4.2. Fluorescence Microscopy

Differentiated glioblastoma cells were seeded in 24-well plates (Sarstedt, Nümbrecht, Germany) with a density of 1500 cells/well, on glass coverslips (Langenbrinck GmbH, Emmendingen, Germany). Cells were fixed with 4% paraformaldehyde at room temperature (RT) for 20 min. The cells were then permeabilized with 20% Methanol for 15 min. To block unspecific binding of antibody, cells were then incubated with 20% Bovine serum albumin (BSA) at RT for 60 min. Primary antibody mix (200 μL; rabbit polyclonal anti-connexin-43 (Sigma, St. Louis, MO, USA) and rabbit monoclonal ß-tubulin (Cell signaling, Cambridge, UK), dilution 1:1000) was added to each coverslip containing wells and incubated at 37 °C for 60 min. Incubation with secondary antibodies (goat anti-rabbit Alexa Fluor 488 and Alexa Fluor 647 (Sigma, St. Louis, MO, USA), dilution 1:1000) was performed at RT for 60 min. DAPI Fluoromount-G^®^ mounting medium (SouthernBiotech, Birmingham, AL, USA) was used for nuclei staining. Images were taken with the use of AX 70 microscope and processed with Zeiss Zen software (Carl Zeiss Microscopy GmbH, Oberkochen, Germany). Fluorescence intensity was calculated as follows: integrated density—(Area of selected cells × mean fluorescence of background).

### 4.3. IDH1 and IDH2 Mutation Analysis

DNA was extracted from glioblastoma cell lines using the QlAamp DNA Mini Kit according to the manufacturer’s standard recommendations (Qiagen, Hilden, Germany). Somatic mutations at codon R132 (IDH1) and R172 (IDH2) were investigated by pyrosequencing as recently described (20). Briefly, IDH1 PCR amplification primers flanking the R132 mutation hotspot of IDH1 were: IDH1-fwd-5′-CACCATACGAAATATTCTGG-3′ and IDH1-rev-biotin-5′- CAACATGACTTACTTGATCC-3′ that amplify 135 bp of genomic DNA. An 87 bp fragment from IDH2 containing the R172 coding region was amplified using the primer set IDH2-fwd-A-5′-AAACATCCCACGCCTAGTCC-3′ and IDH2-rev-5′-biotin-TCTCCACCCTGGCCTACCT-3′. Single-stranded DNA templates were immobilized on streptavidin-coated Sepharose high-performance beads (GE Healthcare, Uppsala, Sweden) using the PSQ Vacuum Prep Tool and Vacuum Prep Worktable (Biotage, Uppsala, Sweden) according to the manufacturer’s instructions, then incubated at 80 °C for 2 min, and allowed to anneal to 0.4 mM sequencing primer IDH1-Py-5′-GTGAGTGGATGGGTAAAACC-3′ at room temperature. IDH2 R172 was sequenced using the pyrosequencing primer IDH2-Py-5′-AGCCCATCACCATTG-3′. Pyrosequencing was performed using PyroGold Reagents (Biotage, Uppsala, Sweden) on the Pyromark Q24 instrument (Biotage, Uppsala, Sweden) according to the manufacturer’s instructions. Pyrogram outputs were analyzed by the PyroMark Q24 software (Biotage, Uppsala, Sweden) using the allele quantification (AQ) software to determine the percentage of mutant versus wild-type alleles. As positive control a glioblastoma sample with confirmed IDH1 mutation or IDH2 mutation as well as negative controls were included.

### 4.4. MGMT Promoter Methylation Sequencing

DNA (500 ng) was subjected to bisulfite conversion using the EpiTect Bisulfite Kit (Qiagen, Hilden, Germany). Bisulfite converted DNA was then analyzed using direct pyrosequencing as described [52]. Briefly, a 266 bp DNA fragment of the MGMT gene was amplified from bisulfite-treated DNA using primers MGMT-forward, 5′-biotin-GGATATGTTGGGATAGTT-3 and MGMT-reverse, 5′-AAACTAAACAACACCTAAA-3. Purification and subsequent processing of the biotinylated single strand DNA was done according to the manufacturer’s instructions. Sequencing was performed on a Pyromark Q24 instrument (Biotage, Uppsala, Sweden) running the CpG assay software (Biotage, Uppsala, Sweden). The primer used for primer extension reaction was 5′-CCCAAACACTCACCAAA-3, which allows sensitive quantification of the methylation status of 8 individual CpG positions, 4 of which have been shown to allow excellent separation between methylated and unmethylated cases or age matched normal brain controls.

### 4.5. Inhibitors and Drugs

Temozolomide (Sigma, St. Louis, MO, USA).

INI-0602 (Wako Pure Chemical Ind., Ltd., Japan).

### 4.6. Determination of Cellular Metabolic Activity

Metabolic activity, as readout for cellular viability, was assessed using a MTT assay as previously described [18]. The tetrazolium dye 3-(4,5-dimethylthiazol-2-yl)-2,5-diphenyltetrazolium bromide (MTT) is reduced to a colored, water-insoluble, formazan by viable cells, which can be spectrophotometrically quantified. Therefore, cells were seeded in a 96-well plate (Sarstedt, Nümbrecht, Germany) with a density of 1500 cells/well. After an incubation period of 24 h, the medium was replaced to contain various concentrations of temozolomide and INI-0602. At the defined measurement time points, the medium was replaced with Phenol red-free medium containing 1 mg/mL MTT salt (Carl Roth, Karlsruhe, Germany) and cells were incubated for 3 h. The reaction was stopped by the addition of Isopropanol and optical density was determined at 550 nm using the µQuant Spectrometer (BioTek Instruments, Winooski, VT, USA).

### 4.7. Cell Viability

Cell viability was assessed using Cell Counting Kit 8 (Abcam, Cambridge, UK). The method is based on the reduction of tetrazolium salt to an orange formazan, which can be detected colorimetrically. The amount of produced formazan is proportional to the number of cells alive. Briefly, cells were seeded in a 384-well plate with a density of 500 cells/well/100 µL and treated 24 h afterwards. After 6 days, 5 µL of the premixed water-soluble tetrazolium salt solution was added into each well. After incubation for 4 h, the absorbance was measured at 460 nm using a microplate reader (Tecan infiinite M200, Zurich, Switzerland).

### 4.8. Cell Confluence Measurement

Fluorescence images were taken every 120 min for 6 days using The IncuCyte^®^ S3 Live-Cell Analysis System (Essen BioScience Inc., Ann Arbor, MI, USA). Briefly, cells were seeded on a 384-well plate (Greiner, Kremsmünster, Austria) with a density of 500 cells/well. After incubation for 24 h, cells were treated with either temozolomide, INI-0602, both or left untreated. The data obtained were processed using the device-specific software (Incucyte 2019B) and cell confluence was determined.

### 4.9. Flow Cytometric Analysis of Cell Death

DNA-fragmentation, as readout for cell death, was assessed by flow cytometric analysis (FACSCanto II, Becton Dickinson, Heidelberg, Germany) of propidium iodide-stained nuclei as previously described [50]. Briefly, cells were seeded into 24-well plates with a density of 9000 cells/well. After incubation for 24 h, the medium was replaced to contain either temozolomide, INI-0602 or a combination of both. At indicated time points, the cells and medium were harvested and centrifuged (5 min, 1300 rpm), resuspended in buffer containing propidium iodide (Sigma, St. Louis, MO, USA) and Triton-X (Sigma, St. Louis, MO, USA), and incubated at 4 °C for 1 h. Each experiment was performed in triplicates and repeated independently three times. The obtained data was analyzed using FlowJo software Version 10.4 (Ashland, OR, USA). Specific DNA-fragmentation was calculated as follows: 100 × (experimental DNA-fragmentation (%) – spontaneous DNA-fragmentation (%))/(100% − spontaneous DNA-fragmentation (%)).

### 4.10. Cell Proliferation Measurement

Cell proliferation was quantified using a BrdU ELISA Kit (Roche, Basel, Switzerland). Briefly, cells were seeded in a 96-well plate with a density of 1,500 cells/well and treated with temozolomide, INI-0602 or both, subsequently. After culturing the cells for 144 h, BrdU labeling solution was added and the cells were reincubated for 2 h. Medium was removed, cells were fixed and DNA was denatured. Then, anti-BrdU-POD solution was added and cells were incubated for additional 1.5 h. A tetramethyl-benzidine solution was added and the BrdU incorporation was determined by measuring the absorbance at 370 and 492 nm.

### 4.11. Reporter Gene Assay

The Cignal™ Finder 45-Pathway Reporter Array (Qiagen, Hilden, Germany) was used to concurrently assess 45 different signaling pathways in glioblastoma cells. For this, cells were seeded into the Reporter Array 96-well plates and reverse transfected with the pathway reporter constructs using Lipofectamine 2000 (Thermo Fisher Scientific, Waltham, MA, USA) according to manufacturers instructions. Each well of the pathway reporter array contained a mixture of an inducible transcription factor responsive construct and constitutively expressing Renilla luciferase construct. Briefly, reporter constructs, present in each plate well, were resuspended with 50 µL Opti-MEM and then mixed with 50 µL diluted (1:100) transfection reagent. Cells were resuspended in Opti-MEM supplemented with 10% of fetal bovine serum and 1% non-essential amino acids (Gibco) at a density of 8e5 cells/mL. Then, 50 µL of the cell suspension was added into each well. After 24 h, the cells were treated as indicated with vehicle (normal growth medium), temozolomide, INI-0602, or both for a period of 3 days. Cells were then lysed and luciferase activities were measured using the Dual-Luciferase^®^ Reporter Assay (Promega, Mannheim, Germany) according to manufacturer’s instructions in a microplate luminometer (Berthold, Germany). Data were normalized for activity of Renilla luciferase to account for transfection efficiency.

### 4.12. Proteome Array

The Human Phospho-Kinase Array (R&D systems, Wiesbaden, Germany) was used to assess the relative phosphorylation level of 43 different kinases in glioblastoma cells. Briefly, cells were seeded with a density of 1e5 cells/mL. After 24 h, cells were treated either with temozolomide, INI-0602 and both or left untreated. At the indicated time points, cell lysates were added to previously blocked nitrocellulose membranes containing 43 different antibodies in duplicate, and incubated overnight at 4 °C on a rocking platform shaker. After three washings steps (10 min each), membranes were incubated with detection antibody mix for 2 h, followed by washing steps (3×) to remove unbound proteins. Then Streptavidin-HRP was applied and incubated at RT for 30 min. Post washing (3×), chemiluminescent detection reagents were added and signal detection was performed using ChemoCam Imager (Intas, Göttingen, Germany). Densitometric quantification was performed using Fiji software Version 2.0 [53].

### 4.13. Protein Immunoblotting

Western blot analysis was performed as previously described [50] using 50 µg of protein from each sample for separation by sodium dodecyl sulphate-gel electrophoresis and following antibodies: rabbit polyclonal anti-Phopsho-JNK (Cell Signaling, Cambridge, UK), rabbit polyclonal anti-JNK (Cell Signaling), rabbit polyclonal anti-Cx43 (Sigma), and mouse anti-ß-Actin (Sigma). Densitometric quantification and normalization to the corresponding beta-actin levels was performed using Fiji software.

### 4.14. Statistical Analysis and Graphing

Statistical analysis was carried out by two-tailed Student’s *t*-test or two-way ANOVA followed by Bonferroni’s post-test. Statistical significance was depicted as * *p* < 0.05, ** *p* < 0.01, *** *p* < 0.001, **** *p* < 0.0001, unless stated otherwise. For graphic processing GraphPad Prism Version 6 (GraphPad Software Inc., La Jolla, CA, USA) and RStudio software Version 1.1 (Boston, MA, USA) was used.

## 5. Conclusions

Inhibition of intercellular communication via gap junctions profoundly sensitizes primary glioblastoma cells to temozolomide-mediated cell death, thereby constituting a promising new therapeutic strategy for patients suffering from this disastrous and currently incurable cancer. Making use of the first gap junction inhibitor optimized for crossing the blood–brain barrier—thus constituting an auspicious drug for clinical applicability—these results may be of significant importance in the course of current translational research in the field of glioblastoma therapy.

## Supplementary Materials

The following are available online at https://www.mdpi.com/2072-6694/11/6/858/s1, Figure S1: Characterization of primary human glioblastoma cell populations, Figure S2: Verification of connexin-43 expression in primary cell population, Figure S3: Determination of effective drug concentrations, Figure S4: The effects on glioblastoma cell viability, Figure S5: Screening for affected pathway-activation.
