# Supplementary material for: Inhibition of Gap Junctions Sensitizes Primary Glioblastoma Cells for Temozolomide
Source: Cancers (Basel). 2019 Jun 20;11(6):858. doi: 10.3390/cancers11060858 (PMC6628126; doi:10.3390/cancers11060858)
Supplement: Supplementary file 1 [file cancers-11-00858-s001.pdf]

Supplementary Materials

Inhibition of Gap Junctions Sensitizes Primary Glioblastoma Cells for Temozolomide

Anna-Laura Potthoff, Dieter Henrik Heiland, Bernd O. Evert, Filipe Rodrigues Almeida, Simon Behringer, Andreas Dolf, Ági Güresir, Erdem Güresir, Ulrich Herrlinger, Kevin Joseph, Torsten Pietsch, Patrick Schuss, Mike-Andrew Westhoff, Hartmut Vatter, Andreas Waha and Matthias Schneider

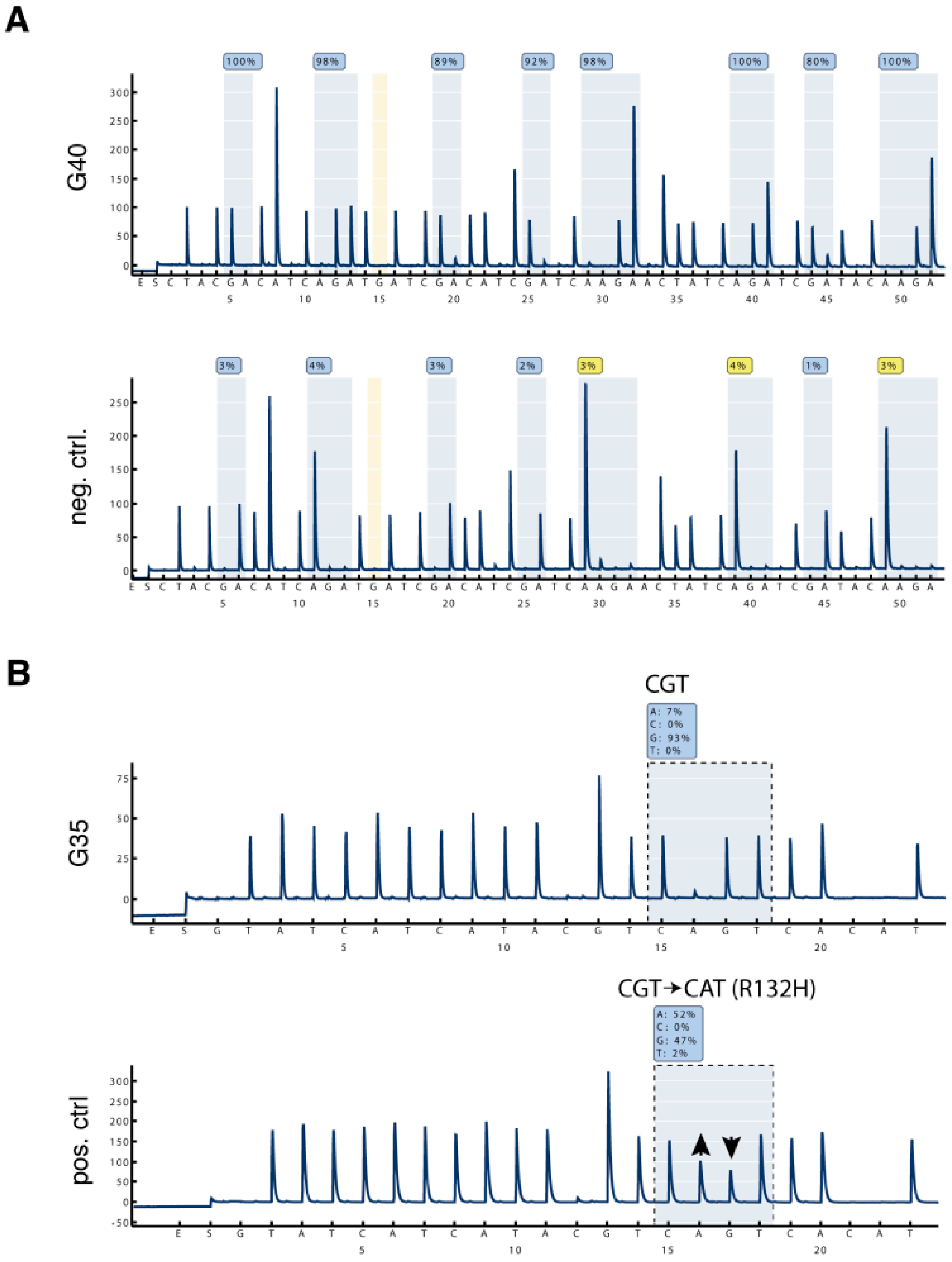

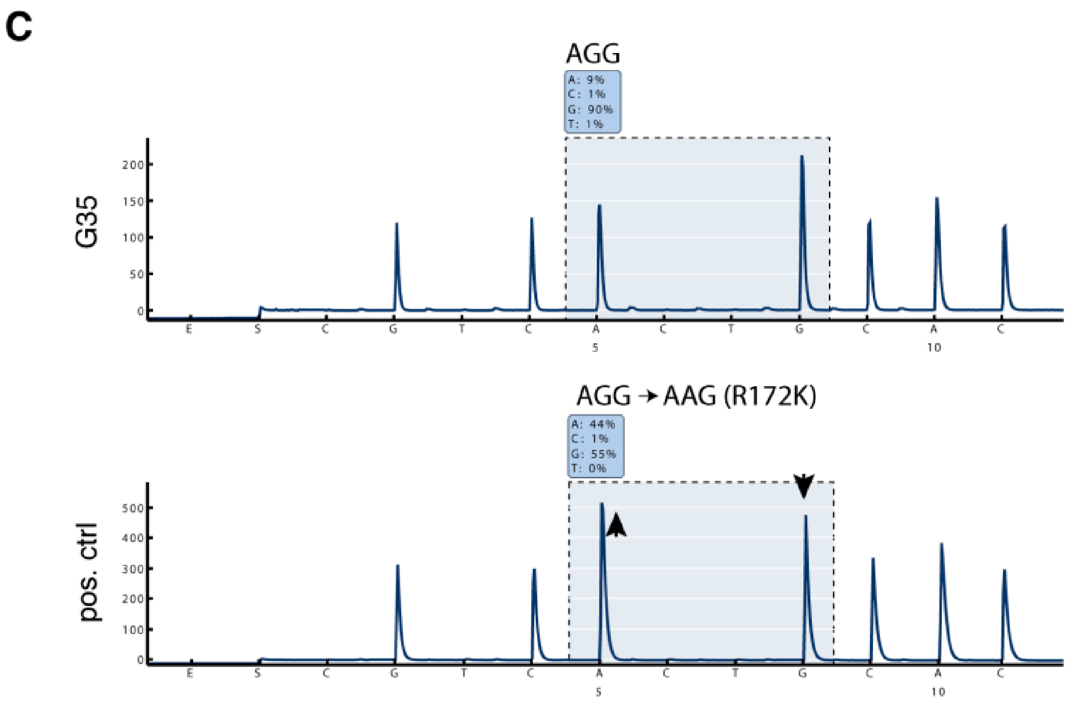

Figure S1. Characterization of primary human glioblastoma cell populations. (**A**) MGMT-methylation analysis: A pyrosequencing assay was used to determine CpG methylation of the MGMT-gene. The reverse assay shows the methylation level at 8 individual CpG positions. A G (guanine) signal in this reverse assay indicates the methylated state of the cytosine of the respective CpG dinucleotide, whereas an A (adenine) signal represents the unmethylated state. Representative for all investigated primary glioblastoma cells, the pyrogram shows strong methylation of the investigated region in the G40 cell population (upper panel). A primary glioma tumor sample serves as negative control (lower panel) and shows the investigated MGMT region in an unmethylated state. (**B**) IDH1- and (**C**) IDH2-mutation analysis: Two DNA fragments spanning the hotspot regions R132 and R172 of IDH1 and IDH2 respectively were amplified by PCR and sequenced by pyrosequencing. The sequence status is depicted for the cell population G35 representative for all three investigated cell populations together with a positive control for the most frequent mutation of the IDH1 (R132H c.395G>A; p.R132H, upper panel) and IDH2 (c.515G>A; p.R172K lower panel). G, glioblastoma.

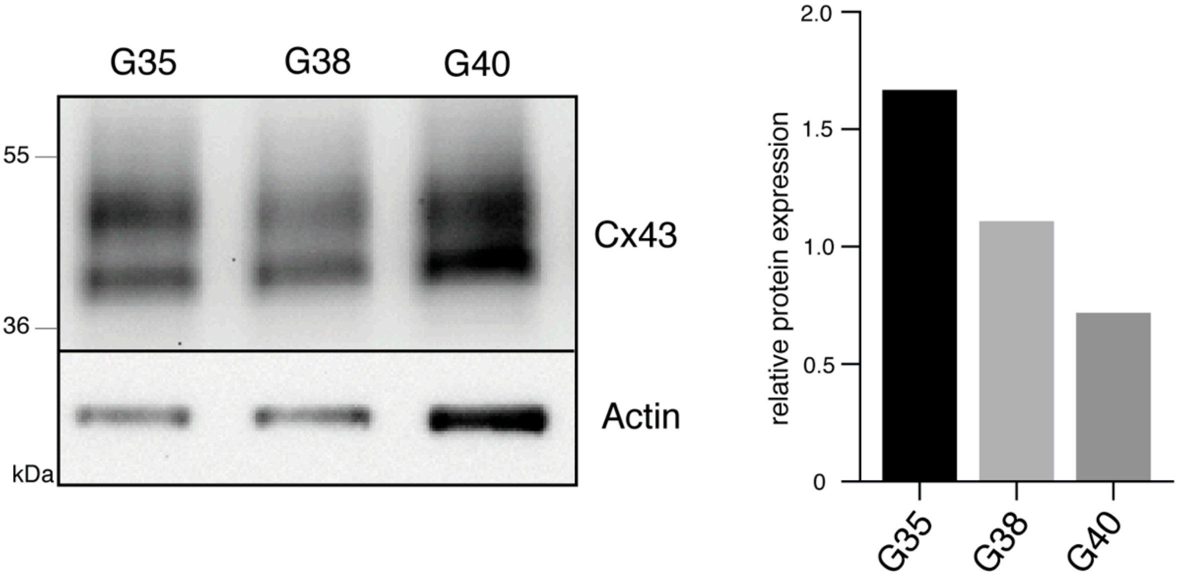

Figure S2. Verification of connexin-43 expression in primary suspension cell population. Western blot analysis and densitometric quantification of Cx43 protein expression: Actin was used as loading

control. Positive connexin-43 expression could also be detected in case of primary suspension cell populations, whereof differentiated glioblastoma cells were derived from for short term expansion. G, glioblastoma; Cx43, connexin-43.

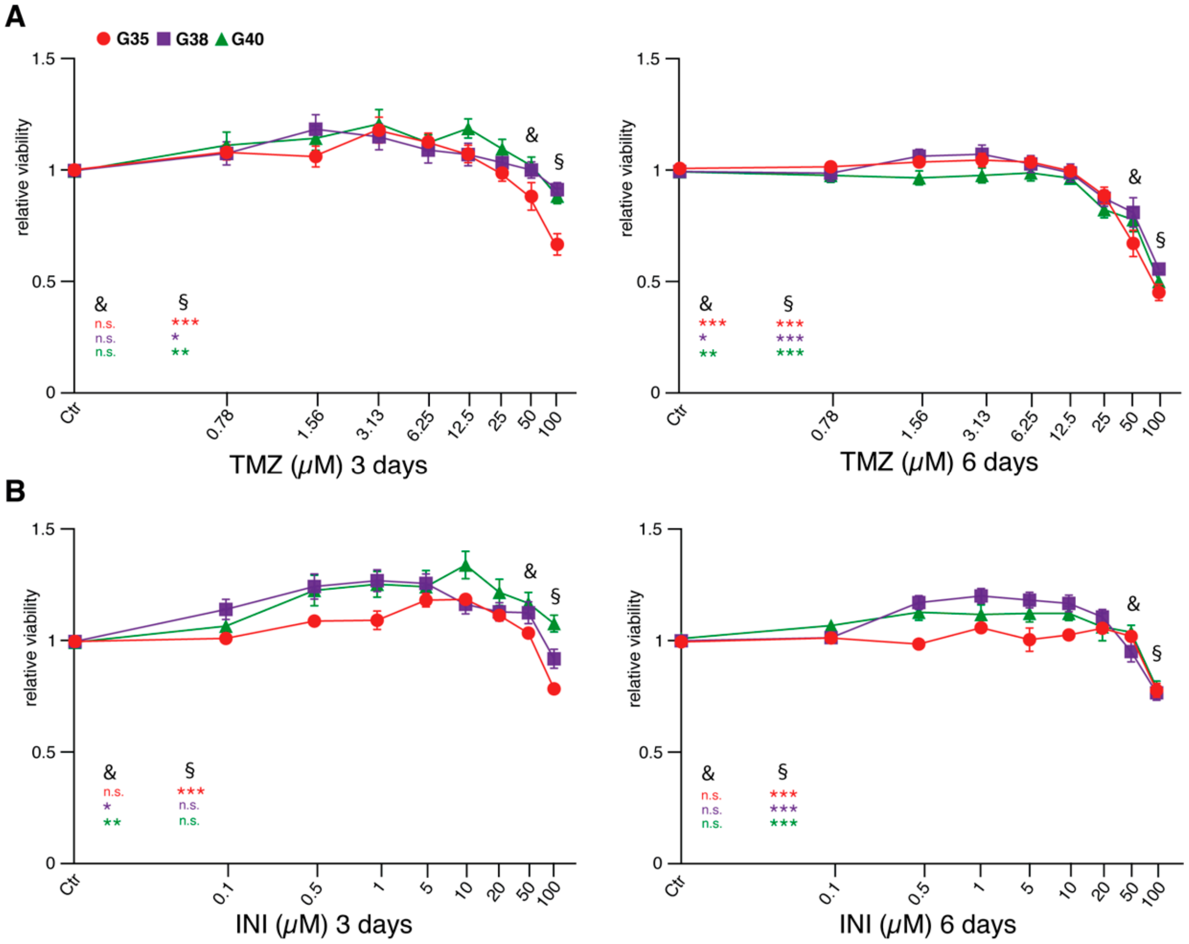

Figure S3. Determination of effective drug concentrations. Results of the MTT assay: Differentiated glioblastoma cells were treated with various concentrations of (A) TMZ and (B) INI. Cell viability was assessed 72 and 144 hrs after treatment. Untreated controls were defined as 100%. Mean of relative cell viability of three independent experiments performed in triplicates are depicted. Error bars show SEM. Ctr, control; G, glioblastoma; TMZ, temozolomide.

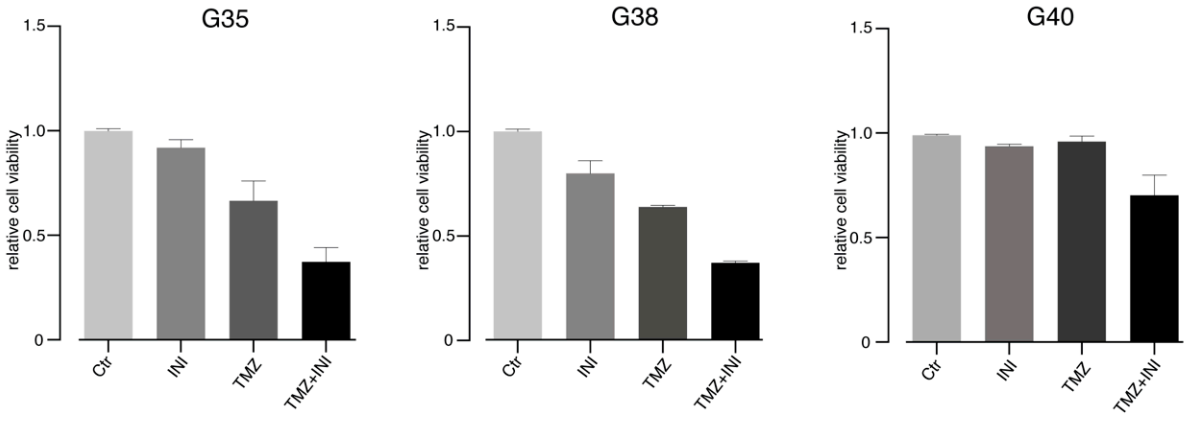

Figure S4. The effects on glioblastoma cell viability. Treatment was performed using 50 μM TMZ, 100 μM INI or combination of both. Reduction of tetrazolium salt to formazan as surrogate readout for cell proliferation was determined colorimetrically 144 hrs after treatment. Untreated controls were defined as 100%. Mean ± SEM is depicted. Ctr, control; G, glioblastoma; TMZ, temozolomide.

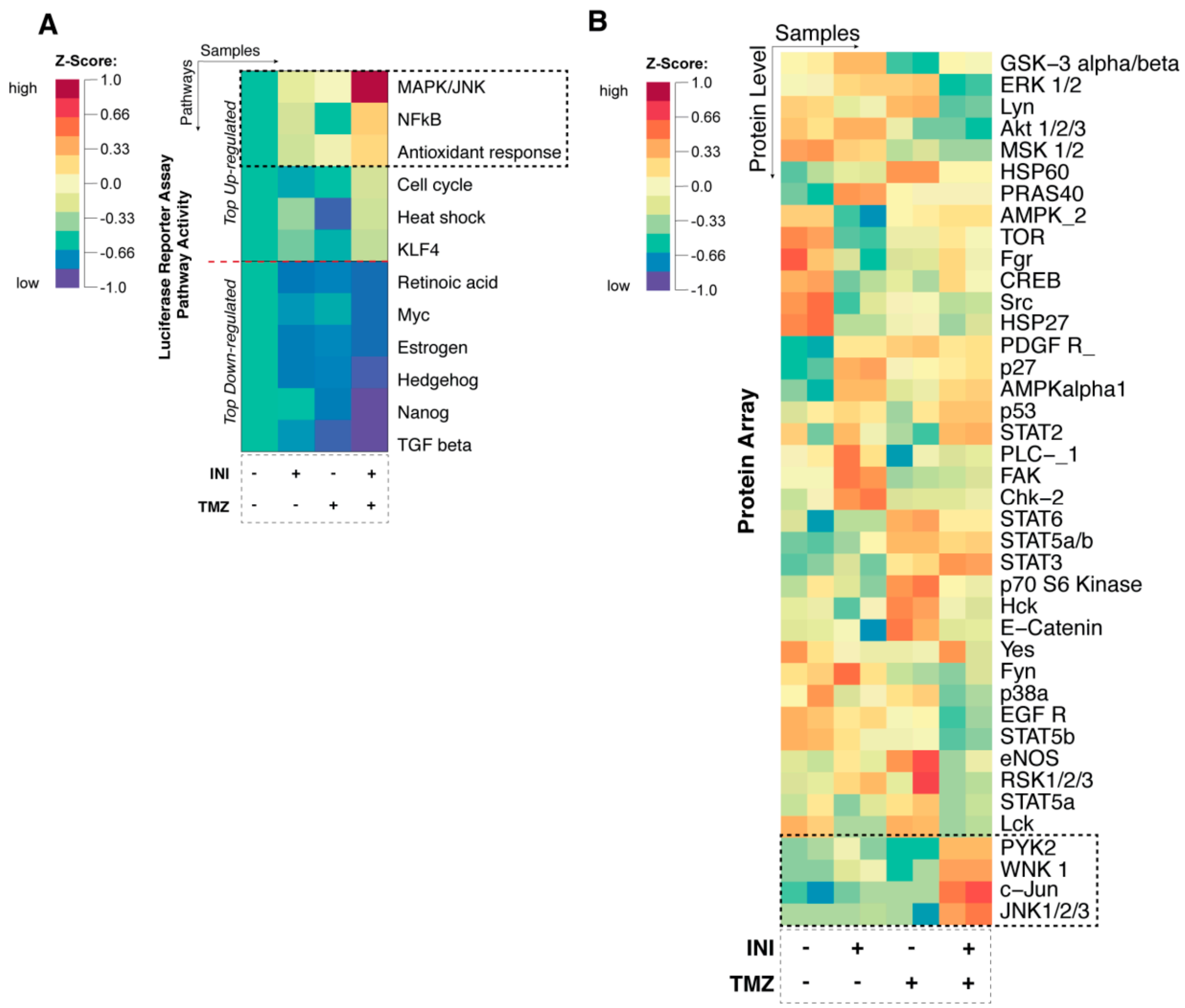

Figure S5. Screening for affected pathway-activation. (**A**) Gene expression heatmap of Cignal™ Finder 45-pathway Reporter Gene Array: Treatment was performed using 50 µM TMZ, 100 µM INI or combination of both. Activity of firefly and Renilla luciferases were measured 72 h after treatment. Data was normalized for activity of Renilla luciferase to account for transfection efficiency. Experiments were performed in duplicates. Results are shown for G35 cell population. (**B**) Heatmap of Proteome Profiler Human Phospho-Kinase Array: Cells were treated with 50 µM TMZ, 100 µM INI or combination of both. Top upregulated transcription factors and protein kinases are depicted. Results are shown for cell population G35. TMZ, temozolomide.

© 2019 by the authors. Licensee MDPI, Basel, Switzerland. This article is an open access article distributed under the terms and conditions of the Creative Commons Attribution (CC BY) license (http://creativecommons.org/licenses/by/4.0/).
